# Slow and steady wins the race: the negative regulators of ethylene biosynthesis in horticultural plants

**DOI:** 10.1093/hr/uhaf108

**Published:** 2025-07-16

**Authors:** Dongdong Li, Shuang Zeng, Ruyi Dai, Kunsong Chen

**Affiliations:** College of Agriculture and Biotechnology, Zhejiang University, Zijingang Campus, Hangzhou 310058, China; Zhejiang Provincial Key Laboratory of Horticultural Plant Integrative Biology, Zhejiang University, Zijingang Campus, Hangzhou 310058, China; The State Agriculture Ministry Laboratory of Horticultural Plant Growth, Development and Quality Improvement, Zhejiang University, Zijingang Campus, Hangzhou 310058, China; College of Agriculture and Biotechnology, Zhejiang University, Zijingang Campus, Hangzhou 310058, China; College of Agriculture and Biotechnology, Zhejiang University, Zijingang Campus, Hangzhou 310058, China; College of Agriculture and Biotechnology, Zhejiang University, Zijingang Campus, Hangzhou 310058, China; Zhejiang Provincial Key Laboratory of Horticultural Plant Integrative Biology, Zhejiang University, Zijingang Campus, Hangzhou 310058, China; The State Agriculture Ministry Laboratory of Horticultural Plant Growth, Development and Quality Improvement, Zhejiang University, Zijingang Campus, Hangzhou 310058, China

## Abstract

The gaseous hormone ethylene controls a variety of physiological processes in horticultural plants, including fruit ripening and elongation, flower development and senescence, and responses to stresses. The functions of ethylene in these processes are intimately linked to its precise biosynthesis, which is finely tuned by a complex network of positive and negative regulators. While significant progress has been made in understanding the roles of positive regulators in ethylene biosynthesis, the negative regulators of ethylene biosynthesis has only recently begun to receive more focus. Ethylene biosynthesis is a simple two-step reaction in land plants, committed by two dedicated enzymes, 1-aminocyclopropane-1-carboxylic acid (ACC) synthase (ACS) and ACC oxidase (ACO). Over the past decade, a growing number of research has identified a wide range of transcriptional, posttranscriptional and epigenetic negative regulators for ACS and/or ACO in horticultural plants, greatly enhancing our understanding of the intricate network that modulates ethylene production. In this review, we provide a comprehensive overview of the negative regulators that mediate ethylene biosynthesis in horticultural plants, with respect to their functions and molecular mechanisms, and their responses to external environmental stimuli or internal growth signals.

## Introduction

**Figure 1:**
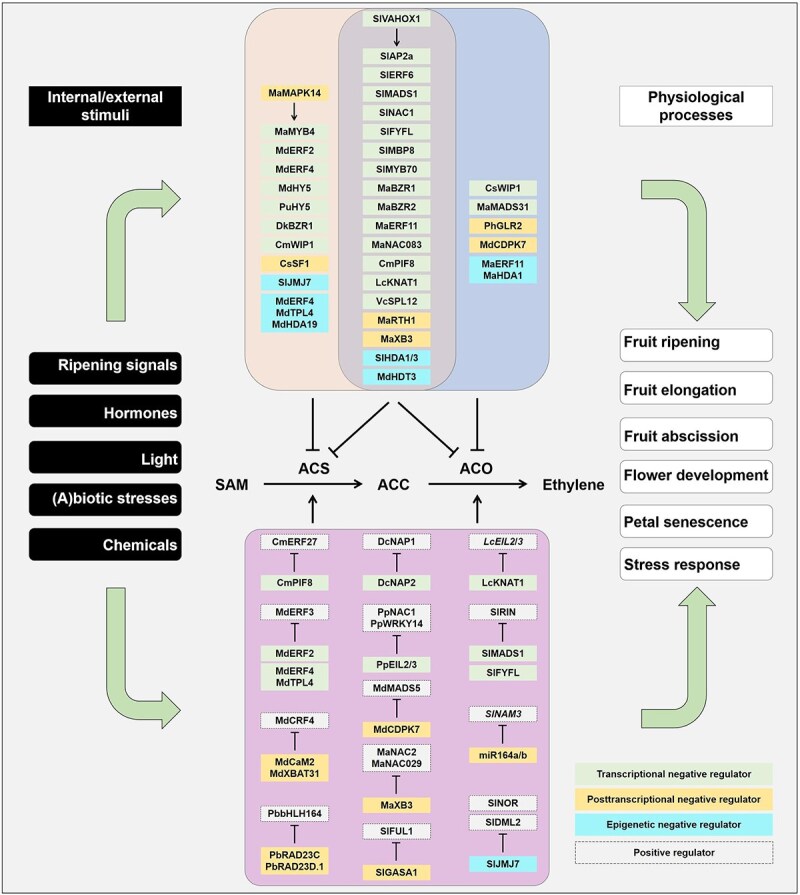
Negative regulators of ACS and/or ACO in the ethylene biosynthesis pathway in horticultural plants. Ethylene biosynthesis in land plants is committed with two steps. In the first step, SAM is converted to ACC by ACS. In the second step, ACC is converted to ethylene by ACO. Transcriptional (in green box), posttranscriptional (in yellow box) and epigenetic (in cyan box) negative regulators directly or indirectly suppress the expression or functions of ACS and/or ACO. They could also function via interfering positive regulators (in gray box) to repress ethylene production. Direct negative regulators include those that directly suppress ACS and ACO, indirect negative regulators include those that inhibit ethylene biosynthesis through the action of direct negative regulators. These negative regulators are involved in physiological processes, including fruit ripening, fruit elongation, fruit abscission, flower development, petal senescence and stress adaption. External and internal cues could have an impact on the fine-tuned regulation of ethylene production. ACC, 1-aminocyclopropane-1-carboxylic acid; ACS, ACC synthase; ACO, ACC oxidase; SAM, *S*-adenosylmethionine.

Ethylene is a simple gaseous molecule that functions as a hormone, playing key roles in a diverse array of plant growth and developmental processes, including germination, seedling growth, flower sexual determination, leaf abscission, and responses to various abiotic and biotic stresses [1]. The ethylene biosynthesis pathway in land plants is mainly a two-step reaction, in which *S*-adenosylmethionine (SAM) is first converted to 1-aminocyclopropane-1-carboxylic (ACC) by ACC synthase (ACS), and ACC then is converted to ethylene by ACC oxidase (ACO; Fig. 1). Although SAM derived from the amino acid methionine by SAM synthetase participates in ethylene biosynthesis, its synthesis diversity and versatile roles in serving as a methyl donor in numerous processes in plant make ACS as the first committed enzyme in the ethylene biosynthesis pathway. Since ACS and ACO are the two enzymes dedicated to ethylene biosynthesis, plant have evolved refined regulatory mechanisms at the transcriptional, posttranscriptional and protein stability levels of these two enzymes to fine-tune ethylene production. This precise regulation ensures that ethylene level align with environmental cues and developmental signals.

In horticultural plants, ethylene production shows dynamic changes, and remaining an optimal amount of ethylene is crucial for many physiological processes. The ethylene biosynthesis can be promoted by various positive regulators, while research in the past decade has identified a growing number of negative regulators in the ethylene biosynthetic pathway in horticultural plants, revealing their unique roles in fine-tuning ethylene biosynthesis. Regarding to ethylene’s double-edged role in many physiological processes, we bring together an overview of the transcriptional, posttranscriptional and epigenetic negative regulators of ACO and ACO. We also discuss their functions across different physiological processes and their responses to external or internal cues. Our focus on the negative regulators of ethylene biosynthesis provides a novel perspective to understand the ‘brakes’ in ethylene biosynthesis.

## Physiological processes that require optimal production of ethylene

In horticultural plants, a number of physiological processes are finely regulated by optimal ethylene production. These include fruit ripening, fruit elongation, fruit abscission, carpel development, flower senescence, and stress responses (Fig. 1). The crucial role of ethylene in fruit ripening has been extensively studied, with significant advances made in identifying positive regulators of ethylene biosynthesis and fruit ripening [2]. However, excessive ethylene production can adversely influence fruit development and alter metabolite profile [3]. Despite the significance of ethylene for fruit development and fruit quality traits, ethylene overproduction can lead to overripe fruit, shorter shelf life, increased decay, and disorders in postharvest fruit [4–6]. From both agricultural and consumer perspectives, the negative regulation of ethylene biosynthesis is of great importance. Indeed, an increasing number of negative regulators of ethylene biosynthesis in fruit has been revealed in recent years. These negative regulators suppress ethylene biosynthesis in unripe fruit, ensuring that ethylene levels remain optimal until the fruit develop a competence for the physiological programs of maturation and ripening.

Optimal control of ethylene biosynthesis is also critical for fruit elongation and flower sex determination. During cucumber fruit development, the elongation of fruit to exogenous ethylene was dosage-dependent, specifically, 10^−1^ ppm of ethylene significantly promoted fruit elongation, whereas 10^0^ and 10^1^ ppm of ethylene inhibited fruit elongation [7]. Reduction of ethylene production by disrupting *CsACS2* strikingly led to shorter fruit than wild type. Consistently, an ethylene overproducing mutant, *cssf1*, also bear shorter cucumber fruit. Moreover, the phenotype could be partially restored by treating with exogenous ACS inhibitor 1-aminoethoxyvinyl glycine [7]. This example the sophisticated regulation of ethylene production in fruit elongation. As intricate as in fruit elongation, the proper production of ethylene determines flower carpel development. In cucumber, a single-nucleotide mutation in CsACO2, the predominant ACO involved in ethylene biosynthesis in primordia, caused monoecious plant conferring androecy [8]. More importantly, treating the androecious mutant with an ethylene supplier induced the development of female flower, while treating ACC did not [8]. The importance of proper ethylene level in determining flower sex has also been well established in cucumber, another Cucurbitaceae species [9]. In both studies, a conserved WIP transcription factor was found to closely regulated ethylene biosynthesis by suppressing the expression of ethylene biosynthetic genes [8, 9].

The production of ethylene is tightly controlled in fruit abscission. Premature fruit abscission causes economic losses, while reducing fruit retention is beneficial for mechanical harvesting. Ethylene is a key hormone in fruit abscission, and extensive studies have shown that ethylene promotes fruit abscission by mediating cell wall remodeling enzymes, such as in apple [10, 11], litchi [12], and sweet cherry [13]. Ethylene biosynthesis in abscission zone can be promoted or suppressed by multiple transcription factors. For instance, in tomato, SlFUL2 enhanced flower pedicel abscission via elevating ethylene production [14]. Conversely, SlFYFL suppressed abscission zone formation, possibly via inhibiting the expression of *SlACS2*, *SlACO1*, and *SlACO3* [15]. In litchi, the transcription factors LcHB2/3 and LcEIL2/3 acted as positive regulators, enhancing the expression of ethylene biosynthetic genes [12, 16]. On the other hand, transcription factor LcKNAT1 negatively regulated ethylene production by both directly suppressing ethylene biosynthetic genes [17] and inhibiting the expression of *LcEIL2*/*3* [18].

Ethylene influences flower opening and more predominantly, it is a key regulator for petal senescence. The modulation of ethylene production is crucial for flower longevity. For instance, it has been shown that exogenous ethylene treatment advanced citrus flower unfolding, while ethylene inhibitors delayed this process [19]. In rose, flower opening was driven by the asymmetric growth of the petal base, and ethylene signaling accelerated this process [20]. However, the induction of ethylene biosynthesis and signaling also rapidly transition flowering into senescence. Many positive regulators of ethylene biosynthesis have been identified in senescent flowers. In petunia, transcription factor PhFBH4 physically bound to the promoter of *PhACS1* and promoted ethylene biosynthesis during flower senescence [21]. Similarly, PhERF71 was a positive regulator for petunia flower senescence and enhanced ethylene production via binding to the promoters of *PhACS4* and *PhACO4* [22]. In carnation, the ethylene downstream signaling master transcription factor DcEIL3-1 could interact with DcWRKY75, another transcription factor that transactivated *DcACS1* and *DcACO1*, promoting ethylene biosynthesis and petal senescence [23]. In peony flower, PlMYB308 activated *PlACO1* expression and positively regulated senescence [24]. Fortunately, the lifespan of flowers was able to be prolonged by negative regulators of ethylene biosynthesis. In petunia flower, PhGLR2 interacted with PhACO1, inhibiting ethylene biosynthesis. Overexpressing PhGLR2 significantly extended flower lifespan [25]. Similarly, carnation petal senescence could be remarkably delayed by overexpressing *DcNAP2*, which suppressed the activation of DcNAP1 on the expression of ethylene biosynthetic genes [26].

Ethylene is an important hormone for plant stress responses, yet its production must be carefully balanced to optimize the trade-off between plant growth and stress reactions. Upon exposure to stresses, the transcript levels of many ethylene biosynthetic genes were leveraged to promote stress tolerance [27]. However, an overproduction of ethylene can have adverse effects on plant growth. For instance, the tomato ethylene overproducing mutant *acs2–1* exhibited earlier seed germination, leaf senescence, altered plant architecture and changes in metabolites [3]. To counteract the adverse effects of excessive ethylene, plants have evolved negative feedback mechanisms that restore ethylene levels to normal after stress, allowing growth to resume. Such regulators include AtARGOs in *Arabidopsis* [28] and NtTCTP in tobacco [29]. When tomato plant underwent cold stress, *Sl-miR164a/b* functioned as a negative feedback regulator for ethylene biosynthesis. *Sl-miR164a/b* suppressed ethylene production by downregulating *SlNAM* transcripts, which was functional in activating ethylene biosynthetic genes. Interestingly, the expression of *Sl-miR164a/b* initially declined sharply in the first few hours in response to cold stress but quickly returned to basal level [30]. Altogether, the function of ethylene in plant is double-edged. Its production requires a finely modulation through a balance between positive and negative regulators. Similar to the regulation of protein degradation rather than *de novo* synthesis in signaling pathways, releasing the inhibitory effect of negative regulators on ethylene biosynthesis in some cases can be a more efficient and prompt way to activate ethylene responses. The identification of emerging negative regulators in ethylene biosynthesis will depict a more comprehensive understanding of how ethylene is controlled in distinct physiological processes in horticultural plants.

## Negative regulators of ACS

ACS is considered as the rate-limiting enzyme in the ethylene biosynthesis pathway. In most land plant species, ACS is encoded by multigene families, with tomato possessing at least nine members. The expression of *ACS* is distinct in different tissues, regulated at the transcriptional level. While ACS proteins also are readily regulated at the posttranslational level [31–33]. ACS protein is not stable and can be rapidly degraded through the 26S proteasome pathway. The stability of ACS protein is tightly regulated by phosphorylation, dephosphorylation, and ubiquitination [33]. Based on the modification sites present in the C-terminus, ACS proteins can be divided into three groups. Type I proteins have both calcium-dependent protein kinases (CDPK) and mitogen-activated protein kinase (MAPK) phosphorylation sites, type II proteins contain one CDPK phosphorylation site and an ubiquitin E3 ligase site, and type III proteins contain no modification site. While the C-terminus of type III ACS proteins lack posttranslational regulatory site, its N-terminal region can be targeted for degradation via ubiquitination by XBAT32 E3 ligase [34]. A recent study identified another E3 ligase, RIE1, which ubiquitinated type III ACS7 in regulating ethylene biosynthesis [35].

A number of transcription factors function as repressors of *ACS* genes (Fig. 1; Table 1). For example, MdERF2 bound to the promoter of *MdACS1* and directly suppressed *MdACS1* expression. Transient antisense *MdERF2* induced *MdACS1* expression, promoted ethylene production and apple fruit ripening. Conversely, overexpressing *MdERF2* inhibited *MdACS1* expression, repressed ethylene production and fruit ripening [36]. *MdACS1* expression was also suppressed by transcription factors MdERF4 and MdHY5 during apple fruit ripening [37, 38]. Negative transcriptional regulators have also been identified in other fruits during ripening. In persimmon, *DkACS1* expression was suppressed by transcription factor DkBZR1 who could physically bind to the brassinosteroid response element in the promoter of *DkACS1* [39]. In banana, DNA affinity purification sequencing combined with RNA-sequencing analyses revealed that MaMYB4 functioned as a suppressor of *MaACS1* expression. Transient overexpressing *MaMYB4* in banana fruit significantly inhibited *MaACS1* expression and ethylene production. Ectopic overexpression of *MaMYB4* in tomato fruit remarkably suppressed the upregulation of *SlACS2* and postponed the ethylene production peak for more than 20 days [40]. During pear postharvest ripening, PuHY5 directly suppressed the expression of *PuACS1*. Transient silencing *PuHY5* promoted fruit ripening, resulting from higher expression of *PuACS1* and ethylene production [41]. Transcriptional negative regulator of ACS was also involved in flower sex determination. *CmACS7* was an important gene in inhibiting stamen development in melon, and CmWIP1 served as its transcriptional suppressor. Loss of function of *CmACS7* led to male flower formation, whereas inactivating CmWIP1 caused strikingly higher *CmACS7* expression, inhibiting stamen development and promoting carpel development, thus generating female flowers [9]. However, the suppression of *CmACS7* expression by CmWIP1 was achieved indirectly, suggesting that co- or intermediary suppressor(s) may exist.

**Table 1:** Negative regulators of ACS.

**Negative regulator**	**Category**	**ACS member**	**Ethylene level altered by regulator compared with control**	**Physiological process**	**Reference**
MdERF2	TF	*MdACS1*	transient antisense, ~200%/overexpressing, ~50%	Fruit ripening	[36]
MdHY5	TF	*MdACS1*	?	Fruit ripening	[37]
MdERF4	TF	*MdACS1*	?	Fruit ripening	[38]
CmWIP1	TF	*CmACS7*(?)	?	Flower development	[9]
DkBZR1	TF	*DkACS1*	Transient overexpressing, ~100%	Fruit ripening	[39]
MaMYB4	TF	*MaACS1*	Transient overexpressing, 50%/ectopic expressing in tomato, delayed	Fruit ripening	[40]
PuHY5	TF	*PuACS1*	Transient silenced, ~160%	Fruit ripening	[41]
CsSF1	E3 ligase	CsACS2	*sf1* mutant, ~150%	Fruit elongation	[7]
SlJMJ7	H3K4 demethylase	*SlACS2*, *SlACS4*	CRISPR/cas9, ~200%/overexpressing, ~50%	Fruit ripening	[42]
MdERF4/MdTPL4/MdHDA19	TF/H3K9 deacetylase	*MdACS3a*	Transient silenced, 160%/overexpressing, 50%	Fruit ripening	[43]

TF, transcription factor. Question mark means not validated or no information. Cm, *Cucumis melo* (melon); Cs, *Cucumis sativus* (cucumber); Dk, *Diospyros kaki* (persimmon); Ma, *Musa acuminata* (banana); Md, *Malus domestica* (apple); Pu, *Pyrus ussuriensis* (pear); Sl, *Solanum lycopersicum* (tomato).

RING-type E3 ligases in *Arabidopsis*, such as ETO1 and XBAT32, functioned as negative regulators of ethylene biosynthesis. They target ACS protein for degradation via the 26S proteasome pathway, and loss-of-function mutants of these E3 ligases produced a significant overproduction of ethylene [34, 44]. ETO1 orthologs exist in many horticultural plant genomes, and ETO1 was capable of interacting with SlACS3. Therefore, attempts have been made to express *ETO1* in tomato fruit to suppress ethylene production during ripening in order to prolong storage life. However, overexpressing *ETO1* did not alter tomato fruit ripening process, because both SlACS2 (type I) and SlACS4 (type III), the two major ACS proteins elevated during fruit ripening, lack the ETO1 ligase ubiquitination site in their C-termini [45]. In contrast, an XBAT E3 ligase, CsSF1, interacted and ubiquitinated CsACS2, playing a significant role in modulating ethylene dosage and controlling cucumber fruit elongation. *csacs2* null mutants bear shorter cucumber fruit and could be restored by exogenous ethylene treatment, suggesting that ethylene promotes cell division [7]. Intriguingly, *CsACS2* expression was stable, but its protein abundance was dynamic during anthesis and fruit development. Further evidence demonstrated that CsACS2 was an ubiquitination substrate of CsSF1 [7]. As a type III ACS, the N-terminal region of CsACS2 likely possesses a ubiquitination site for CsSF1. The *cssf1* loss-of-function mutant had approximately 50% greater ethylene production, but surprisingly, it also bears shorter fruit. It was shown that exogenous application of the ethylene inhibitor 1-aminoethoxyvinyl glycine (AVG) partially rescued the short-fruit phenotype in *sf1* [7]. It collectively shows that ethylene production, mediated via ACS ubiquitination, must be finely tuned to an optimal level for proper fruit elongation.

In addition to transcriptional regulation and ubiquitination, epigenetic negative regulators are also effective in suppressing *ACS* gene expression and ethylene production (Table 1). These negative regulators include histone demethylase and deacetylase. SlJMJ7, a H3K4 demethylase, functioned as a suppressor of ethylene biosynthesis during tomato fruit ripening. Overexpressing or knocking out *SlJMJ7* suppressed ~50% or promoted ~100% of ethylene production compared with wild-type fruit, respectively [42]. Transcriptomic, chromatin immunoprecipitation (ChIP)-PCR, and further ChIP-qPCR analyses demonstrated that SlJMJ7 bound to the gene body of *SlACS2* and *SlACS4*, altering their histone methylation levels. Since H3K4 methylation is typically associated with gene activation, SlJMJ7 directed the demethylation of *SlACS2* and *SlACS4*, thus suppressing their expression [42]. *ACS* expression can also be regulated by histone acetylation. Histone acetylation provides a more accessible chromatin structure and is also generally associated with enhanced gene expression [46]. MdHDA19, a histone H3K9 deacetylase, was shown to regulate ethylene production in apple fruit. Transient silencing or overexpressing *MdHDA19* led to ~60% greater or ~ 50% reduced ethylene production in apple fruit, respectively. It was found that MdERF4/MdTPL4/MdHDA9 complex decreased the H3K9 acetylation level in the gene body of *MdACS3a* resulting in its lower expression. This negative complex for *MaACS3a* expression was attenuated by a C-G mutation in the repression motif of MdERF4, which resulted in higher histone acetylation and increased expression of *MdACS3a* [43].

## Negative regulators of ACO

ACO is the ultimate enzyme that converts the precursor ACC into ethylene in seed plants, making the regulation of *ACO* expression and activity particularly important. ACO is a member of the 2-oxoglutarate-dependent dioxygenase superfamily, encoded by multigene families in most plants [47]. *ACO* genes can be suppressed by transcription factors (Fig. 1; Table 2). For instance, CsWIP1, whose homolog CmWIP1 in melon has been mentioned previously, could directly bind to the promoter of *CsACO2* and repressed its expression. This negative regulation was crucial for cucumber flower sex determination [8]. During banana fruit ripening, transcription factor MaMADS31 suppressed the expression of *MaACO13* by directly recognizing the CArG-box element in its promoter. Transient overexpressing *MaMADS31* inhibited *MaACO13* expression and postponed the peak of ethylene production [48].

**Table 2:** Negative regulators of ACO.

**Negative regulator**	**Category**	**ACO member**	**Ethylene level altered by regulator compared with control**	**Physiological process**	**Reference**
CsWIP1	TF	*CsACO2*	?	Flower development	[8]
MaMADS31	TF	*MaACO13*	Transient overexpressing, delayed	Fruit ripening	[48]
PhGLR2	Membrane protein	PhACO1	TRV-based silencing, ~250%/Overexpressing, ~50%	Petal senescence	[25]
MdCDPK7	Kinase	MdACO1	Transient antisense, ~200%	Fruit ripening	[49]
MaERF11/MaHDA1	TF/histone deacetylase	*MaACO1*	?	Fruit ripening	[50]

TF, transcription factor. Question mark means not validated or no information. Cs, *C. sativus* (cucumber); Ma, *M. acuminata* (banana); Md, *M. domestica* (apple); Ph, *Petunia hybrida* (petunia).

ACO can also be modulated at the posttranslational level (Table 2). In tomato, *S*-sulfhydration of SlACO1 and SlACO2 has been shown to inhibit ACO activity [51], while *S*-nitrosylation of SlACO4h promoted ACO activity [52]. However, the specific regulators controlling these posttranslational modifications are yet to be identified. In *Arabidopsis*, REVERSION-TO-ETHYLENE SENSITIVITY1 (RTE1) modulated ethylene response via interacting with ethylene receptor ETR1 [53]. Intriguingly, PhGLR2, the closest homolog of AtRTH (RTE1 homolog) in petunia, directly interacted with PhACO1 and inhibited its activity in oxidizing ACC into ethylene [25]. Silencing *PhGLR2* significantly promoted ethylene production and accelerated flower senescence, whereas overexpressing *PhGLR2* reduced ethylene production and extended flower longevity [25]. More recently, a study showed that MdACO1 was phosphorylated by MdCDPK7, leading MdACO1 protein to be degraded via the 26S proteasome pathway. Transient silencing of *MdCDPK7* promoted ethylene production and apple fruit ripening [49].

*ACO* gene expression is also epigenetically regulated by histone deacetylase (Table 2). In unripe banana fruit, MaERF11 inhibited *MaACO1* expression by recruiting histone deacetylase MaHDA1. During ripening, MaERF11 level was reduced to release the inhibitory impact of MaERF11-MaHDA1 complex on *MaACO1* expression, thus resulting in increased ethylene production [50]. Altogether, a growing number of negative regulators of ACO have been identified. Nevertheless, the detailed molecular mechanisms with respect to *ACO* expression inhibition and protein degradation remain to be fully uncovered.

## Co-negative regulators of ACS and ACO

Instead of sorely suppressing ACS or ACO, a large number of negative regulators simultaneously inhibit both enzymes (Fig. 1; Table 3). These negative regulators are primarily involved in fruit ripening, which is expected since ethylene plays a major role in fruit ripening, and limiting ethylene production is crucial for fruit shelf life. Among these regulators, some may function more as ripening inhibitors than ethylene biosynthesis suppressors. Although transient overexpressing these negative regulator genes repressed ethylene production while silencing them led to overproduction of ethylene, they may indirectly influence the expression of *ACS* or *ACO* genes since no evidence were provided to establish that they are direct suppressors of *ACS* or *ACO*. These negative regulators include transcription factors such as SlAP2, SlERF6, SlMADS1, SlFYFL, and SlMBP8, as well as histone deacetylases like SlHDA1 and SlHDA3 (Table 3). In banana fruit, albeit transient *MaRTH1* reduced expression of *MaACS1* and *MaACO1*, and ethylene production decreased to be ~80% of that in control fruit, MaRTH1 may interfering with ethylene receptor for ethylene signaling response [70]. Another example is SlE8 in tomato fruit. *SlE8* was highly expressed in ripening fruit and shared similarity in amino acid sequence with ACO. Antisense or co-suppressing *SlE8* led to remarkably high ethylene levels in transgenic fruit, suggesting that SlE8 is an ethylene biosynthesis negative regulator [67, 68]. However, the mechanism by which SlE8 represses ethylene production in tomato fruit remains unclear. Although ACO proteins can form heterodimers, *in vitro* assays showed that SlE8 did not alter SlACO1 activity [74]. It remains to be tested that whether SlE8 protein physically interacts and inhibits ripening-related regulators. In 2021, a study found that *SlE8* encoded an enzyme that catalyzed the C-27 hydroxylation reaction in the α-tomatine metabolic pathway, which metabolized the bitter steroidal glycoalkaloids α-tomatine into non-bitter esculeoside A [75]. It remains unclear whether the depletion of certain metabolite(s) by the metabolic function of SlE8 could suppress ethylene biosynthesis or fruit ripening signaling.

**Table 3:** Co-negative regulators of ACS and ACO.

**Negative regulator**	**Category**	**ACS and ACO member**	**Ethylene level altered by regulator compared with control**	**Physiological process**	**Reference**
SlAP2a	TF	*ACS2*, *ACS4*, *ACO1* (?)	RNAi, ~400 to 500%	Fruit ripening	[54]
SlAP2a	TF	*SAMS3*, *ACS6*, *ACO4* (?)	RNAi, ~300 to 400%	Fruit ripening	[55]
SlERF6	TF	*SlACS2*, *SlACO1*, *SlACO3* (?)	RNAi, ~200%	Fruit ripening	[56]
SlMADS1	TF	*SlACS1a*, *SlACS2*, *SlACS6*, *SlACO1*, *SlACO3* (?)	RNAi, ~300%	Fruit ripening	[57]
SlNAC1	TF	*SlACS2*, *SlACO1*	Overexpressing, ~50%	Fruit ripening	[58]
SlFYFL	TF	*SlACS2*, *SlACO1*, *SlACO3*(?)	Overexpressing, ~50%	Fruit ripening Fruit abscission	[15]
SlMBP8	TF	*SlACS2*, *SlACO1*, *SlACO3* (?)	RNAi, ~150%	Fruit ripening	[59]
SlMYB70	TF	*SlACS2*, *SlACO3*	RNAi, ~300% /overexpressing, ~50%	Fruit ripening	[60]
MaERF11	TF	*MaACS1*, *MaACO1*	?	Fruit ripening	[61]
MaBZR1/2	TF	*MaACS1*, *MaACO13*, *MaACO14*	?	Fruit ripening	[62]
MaNAC083	TF	*MaACS1*, *MaACO1*, *MaACO4*, *MaACO5*, *MaACO8*	Transient silenced, accelerated/transient overexpressing, delayed	Fruit ripening	[63]
LcKNAT1	TF	*LcACS1*, *LcACS7*, *LcACO2*, *LcACO3*	?	Fruit abscission	[17]
CmPIF8	TF	*CmACS10*, *CmACO1*	Transient silenced, 150%	Fruit ripening Stress response	[64, 65]
VcSPL12	TF	*VcACS1, VcACO6*	Transient silenced, 233% in ACC content/overexpressing, 44% in ACC content	Fruit ripening	[66]
SlE8	Enzyme protein	*SlACS2*, *SlACO1* (?)	Antisense, ~200% to ~700%/co-suppress, ~500%	Fruit ripening	[67, 68]
MaXB3	E3 ligase	MaACS1, MaACO1	transient overexpressing, ~ 33%/ectopic expression, ~33%	Fruit ripening	[69]
MaRTH1	Membrane protein	*MaACS1*, *MaACO1* (?)	Transient overexpressing, ~80%	Fruit ripening	[70]
SlHDA1	Histone deacetylase	*SlACS2*, *SlACS4*, *SlACO1*, *SlACO3* (?)	RNAi, ~140%	Fruit ripening	[71]
SlHDA3	Histone deacetylase	*SlACS2*, *SlACS4*, *SlACO1*, *SlACO3* (?)	RNAi, ~140%	Fruit ripening	[72]
MdHDT3	Histone deacetylase	*MdACS1*, *MdACO1*	Transient overexpressing, ~60%	Fruit ripening	[73]

TF, transcription factor. Question mark means not validated or no information. Cm, *C. melo* (melon); Lc, *Litchi chinensis* (litchi); Ma, *M. acuminata* (banana); Md, *M. domestica* (apple); Sl, *S. lycopersicum* (tomato); Vc, *Vaccinium corymbosum* (blueberry).

Some transcription factors can suppress both *ACS* and *ACO* expression during fruit ripening. In tomato, SlNAC1 or SlMYB70 directly suppressed the expression of *SlACS2* and *SlACO1* or *SlACO3*, respectively [58, 60]. In blueberry, VcSPL12 could bind to the promoters of *VcACS1* and *VcACO6* to inhibit ethylene production and anthocyanin biosynthesis [66]. In melon, CmPIF8 bound to *CmACS10* and *CmACO1* promoters to inhibit their expression [64, 65]. The number of target genes in the ethylene biosynthesis pathway that could be suppressed by one single transcription factor is variable, in some cases, it can target up to five genes simultaneously. For example, MaERF11 suppressed two ethylene biosynthesis genes, *MaACS1* and *MaACO1* [61]; MaBZR1 and MaBZR2 inhibited the expression of three genes, *MaACS1*, *MaACO13*, and *MaACO14* [62]; LcKNAT1 suppressed four ethylene biosynthetic genes, *LcACS1*, *LcACS7*, *LcACO2*, and *LcACO3* [17]; and MaNAC083 targeted and suppressed five ethylene biosynthesis genes, *MaACS1*, *MaACO1*, *MaACO4*, *MaACO5*, and *MaACO8* [63]. It is particularly fascinating to understand how these negative regulators, such as those identified in banana, coordinate among themselves to control ethylene production.

Both MaACS1 and MaACO1 can be recognized by an E3 ligase, MaXB3, for ubiquitination and degradation during banana fruit ripening [69]. Transient overexpressing *MaXB3* caused substantial less abundances of MaACS1 and MaACO1 proteins, suppressing ethylene production. Ectopically expressing *MaXB3* in tomato also repressed ethylene production and fruit ripening [69]. Notably, to our knowledge, MaXB3 is currently the only E3 ligase identified involved in ACO ubiquitination and degradation. Whether MaXB3 orthologs in other plant species are conserved in ubiquitinating ACO, and whether other E3 ligases can also ubiquitinate ACO, await to be explored.

**Table 4:** Indirect negative regulators by enhancing the suppression of direct negative regulators on ethylene biosynthesis.

**Negative regulator**	**Category**	**Enhanced negative regulator**	**Ethylene level altered by regulator compared with control**	**Physiological process**	**Reference**
SlVAHOX1	TF	*SlAP2a*	RNAi, ~130%/Overexpressing, 80%	Fruit ripening	[76]
MaMAPK14	Kinase	MaMYB4	Transient overexpressing, 50%	Fruit ripening	[77]

TF, transcription factor. Ma, *M. acuminata* (banana); Sl, *S. lycopersicum* (tomato).

**Table 5:** Indirect negative regulators that interfering the functions of direct positive regulators of ethylene biosynthesis.

**Negative regulator**	**Category**	**Positive regulator**	**Target ethylene biosynthetic gene**	**Physiological process**	**Reference**
SlMADS1	TF	SlRIN	*SlACS2*, *SlACO1* etc.	Fruit ripening	[57]
SlFYFL	TF	SlRIN	*SlACS2*, *SlACO1* etc.	Fruit ripening Fruit abscission	[15]
MdERF2	TF	MdERF3	*MdACS1*	Fruit ripening	[36]
MdERF4/TPL4	TF	*MdERF3*	*MdACS1*	Fruit ripening	[79]
LcKNAT1	TF	*LcEIL2*/*LcEIL3*	*LcACS1*, *LcACS7*, *LcACO2*, *LcACO3*	Fruit abscission	[18]
DcNAP2	TF	DcNAP1	*DcACS1*, *DcACO1*	Petal senescence	[26]
PpEIL2/3	TF	*PpNAC1*/PpWRKY14	*PpACS1*, *PpACO1*	Fruit ripening	[80]
CmPIF8	TF	CmERF27	*CmACS10*	Stress response	[65]
MaXB3	E3 ligase	MaNAC2	*MaACS1*, *MaACO1*	Fruit ripening	[69]
MaXB3	E3 ligase	MaNAC029	*MaACS1*, *MaACO1*, *MaACO13*	Fruit ripening	[81]
Sl-miR164a/b	miRNA	*SlNAM3*	*SlACS1a*, *SlACS1b*, *SlACO1*, *SlACO4*	Stress response	[30]
SlGASA1	Small peptide	SlFUL1	*SlACS2*, *SlACO1*	Fruit ripening	[82]
MdCaM2/MdXBAT31	Calmodulin/E3 ligase	MdCRF4	*MdACS1*	Fruit ripening	[83]
MdCDPK7	Kinase	MdMADS5	*MdACS1*	Fruit ripening	[49]
PbRAD23C/D.1	Ubiquitin complex transporter	PbbHLH164	*PbACS1b*	Fruit ripening	[84]
SlJMJ7	H3K4 demethylase	*SlRIN*, *SlNOR*, *SlDML2*	*SlACS2*, *SlACS4*, *SlACO6* etc.	Fruit ripening	[42]

TF, transcription factor. Cm, *C. melo* (melon); Dc, *Dianthus caryophyllus* (carnation); Lc, *L. chinensis* (litchi); Ma, *M. acuminata* (banana); Md, *M. domestica* (apple); Pb, *Pyrus bretschneideri* (pear); Pp, *Prunus persica* (peach); Sl, *S. lycopersicum* (tomato).

A very recent study found that MdHDT3 directly bound to the promoters of *MdACS1* and *MdACO1*, negatively regulating ethylene production in apple fruit by modulating the acetylation levels of these two genes [73]. This study underscores the importance of epigenetic negative regulators of *ACS* and *ACO* in balancing ethylene biosynthesis.

## Indirect negative regulators by enhancing the suppression of direct negative regulators on ethylene biosynthesis

Some regulators suppress ethylene biosynthesis indirectly through enhancing the expression or activity of those direct negative regulators (Fig. 1; Table 4). As for transcription factor, it activates the expression of direct negative regulator. For example, RNAi silencing a HD-zip I transcription factor, *SlVAHOX1*, accelerated tomato fruit ripening, and overexpressing *SlVAHOX1* inhibited ripening. Among the differentially expressed genes from transcriptome analysis, the expression of *SlAP2a*, a known ethylene biosynthesis negative regulator, was significantly downregulated in *SlVAHOX1*-RNAi fruit. Further analyses revealed that SlVAHOX1 activated the expression of *SlAP2a* by physically binding to its promoter [76]. As for protein kinase, it phosphorylates negative regulator to enhance the inhibitory function on ethylene biosynthesis. In banana fruit, MaMAPK14 was found to phosphorylate MaMYB4, a direct negative regulator of *MaACS1*. The phosphorylation of MaMYB4 strengthened the transcriptional repression on *MaACS1*, elevated its binding capacity and protein stability [77]. Protein phosphorylation is also critical for ACS protein stability and ethylene production. In *Arabidopsis*, ACS6 was phosphorylated by MAPK6 to prevent from degradation [78]. Therefore, the identification of MaMAPK14 as a negative regulator for ethylene production in banana fruit expands our understanding of MAPK signaling cascade in fine-tuning the production of ethylene by differentially regulating ethylene biosynthetic enzymes.

## Indirect negative regulators that interfering the functions of direct positive regulators of ethylene biosynthesis

There are a variety of negative regulators controlling ethylene production by disrupting the functions of positive regulators (Fig. 1; Table 5). Some of these negative regulators are transcription factors that physically bound to the promoters and suppressed the expression of positive regulators. For example, in apple fruit ripening, MdERF4/TPL4 suppressed the transcription of *MdERF3*, a transcription factor that activated *MdACS1* expression, thereby repressing ethylene production [79]. In litchi fruit abscission, LcEIL2/3 were master transcription factors in the ethylene signaling pathway, promoting a set of ethylene biosynthesis genes. It was identified that LcKNAT1 negatively controlled ethylene production by directly inhibiting the expression of *LcEIL2*/*3* [18]. By contrast, PpEIL2/3 suppressed the expression of *PpNAC1*, a positive regulator of ethylene biosynthesis in peach fruit ripening [80]. In some cases, transcription factors played negative roles in ethylene biosynthesis via interacting with and reducing the transcriptional activity of positive regulators. In tomato, SlRIN was a key MADS transcription factor that exaggerated ethylene biosynthesis [85]. While two other MADS transcription factors, SlMADS1 and SlFYFL, interacted with SlRIN, possibly interfering the transcriptional activity of SlRIN and thus playing negative functions in ethylene biosynthesis [15, 57]. In carnation, the NAC transcription factor DcNAP1 promoted flower petal senescence through binding to the promoters of *DcACS1* and *DcACO1*, and activating their expression to induce ethylene biosynthesis. An insertion of a dTdic1 transposon in the exon of *DcNAP1* led to alternative splicing which transcribed a second variant, DcNAP2. DcNAP2 interacted with DcNAP1 and inhibited the transcriptional activation of DcNAP1 on ethylene biosynthesis genes, thus suppressing ethylene biosynthesis [26]. A similar molecular mechanism was observed in melon, where the interaction between CmPIF8 and CmERF8 reduced ethylene biosynthesis, involving in powdery mildew resistance [65]. In apple fruit, MdERF2 inhibited ethylene production by joint effects from transcriptional suppression and protein–protein interaction. MdERF3 positively transactivating *MdACS1* expression to promote ethylene production. MdERF2 directly suppressed the expression of *MdERF3* by promoter binding. MdERF2 also interacted with MdERF3 to reduce its activity in promoting *MdACS1* expression. Collectively, transcription factors are a majority of negative regulators for ethylene production, functioning through transcription inhibition and protein–protein interfering of positive regulators.

Through protein–protein interaction, a small peptide, SlGASA1, also negatively regulated ethylene biosynthesis in tomato fruit ripening. SlGASA1 was found to interact with SlFUL1, a key ripening regulator that has been extensively studied [86]. The interaction between SlGASA1 and SlFUL1 repressed the upregulation of *SlASC2* and *SlACO1* [82].

**Table 6:** The endogenous metabolism molecules that suppress ethylene biosynthesis.

**Molecule**	**Species**	**Target and impact**	**Physiological process**	**Reference**
H_2_S	Tomato	SlACO1 and SlACO2 protein underwent persulfidation and reduced activity; H_2_S also downregulated the expression of *SlACO*s	Fruit ripening	[51, 90]
Methylglyoxal (MG)	Tomato	Potential methionine or SAM biosynthesis enzymes, MG may glycate and suppress enzymatic activity	Fruit ripening	[91]

Some regulators negatively control ethylene biosynthesis by leading to the ubiquitination and degradation of positive regulators. In banana, the E3 ligase MaXB3 targeted two transcription factors, MaNAC2 and MaNAC029, both of which were involved in promoting fruit ripening. By ubiquitinating these factors, MaXB3 played a negative role in the regulation of ethylene biosynthesis [69, 81]. Similarly, in apple and pear fruit ripening, MdCaM2/MdXBAT31 and PbRAD23C/D.1 functioned in the ubiquitination of key positive regulators, thus negatively mediating ethylene biosynthesis [83, 84].

In apple, MdACO1, a key enzyme in ethylene biosynthesis, can be phosphorylated by MdCDPK7, leading to its degradation via the proteasome pathway. This phosphorylation process of MdCDPK7 also extended to MdMADS5, a transcriptional activator of *MdACS1*, which was also a key gene in ethylene production in apple fruit. Phosphorylation by MdCDPK7 led to the degradation of MdMADS5, further reducing ethylene biosynthesis [49]. These findings present a dual role of CDPK in regulating ethylene production, both directly through ACO protein and indirectly through transcriptional activator of *ACS*.

microRNAs (miRNAs) are an extensive class of short nucleotides that posttranscriptionally regulate gene expression in plants [87]. A study revealed that miRNA also functioned as negative regulator of ethylene biosynthesis in tomato plant when exposed to cold stress. The transcription factor SlNAM3 positively participated in cold tolerance by directly enhancing expression of ethylene biosynthetic genes, *SlACS1a*, *SlACS1b*, *SlACO1,* and *SlACO4*. Its expression was found to be inhibited by Sl-miR164a/b, and silencing *Sl-miR164a/b* elevated *SlNAM3* expression and ethylene production under cold treatment [30]. In a distinct manner, a recent study found that a long non-coding RNA could bind to SAMS transcripts and promote biosynthesis of ethylene precursors [88]. The regulation of ethylene biosynthesis genes at the posttranscriptional level is complicated. Although miRNA and long non-coding RNA have been predicted to target ethylene biosynthesis genes in tomato (*SlACO2* was potentially targeted by MSTRG.59396.1 and miR396b) [89], their biological and physiological functions have not been well understood.

Epigenetic enzyme can also suppress ethylene biosynthesis by inhibiting the expression of key ethylene biosynthesis regulators. In tomato fruit, SlJMJ7, a H3K4 demethylase, negatively modulated the expression of *SlRIN*, *SlNOR*, and *SlDML2*, three well established ripening regulators, thus achieving an overwhelming inhibition on ethylene production during ripening [42].

## Endogenous metabolism molecules that suppress ethylene biosynthesis

Several endogenous metabolites and signaling molecules functioned as negative regulators of ethylene biosynthesis, with effects ranging from altering enzyme activity to modulating gene expression (Table 6). A study found that disruption of a D-cysteine desulfhydrase, SlDCD2, promoted tomato fruit ripening and ethylene production [90]. This negative influence on ethylene production was attributed to the reduction of hydrogen sulfide (H_2_S) production and resulting accumulation of H_2_O_2_ and malondialdehyde [90]. Another study showed that osmotic stress increased endogenous H_2_S content in tomato plant. H_2_S precursor NaHS treatment inhibited ACO activity and ethylene production. Heterologously expression and persulfidation assays showed that NaHS caused persulfidation of LeACO1 and LeACO2 in a dose-dependent manner [51]. Liquid chromatography–tandem mass spectrometry analysis further revealed that Cys60 in LeACO1 was the persulfidation site. A mutation of cysteine to serine in LeACO1 did not alter its basal ACO activity but abolished the persulfidation and activity inhibition of LeACO1 by NaHS [51]. These results revealed that H_2_S molecule directly suppressed ethylene biosynthesis by persulfidating specific amino acid in ACO protein and reducing its enzyme activity. In addition, H_2_S could downregulate expression of *LeACO*s via an ethylene dependent or independent pathway to suppress ethylene biosynthesis in tomato plant [51]. In contrast, another important gaseous signaling molecule, nitric oxide (NO), promoted ethylene biosynthesis by *S*-nitrosylation on ACO protein. NO content was induced by salt stress in tomato plant. NO donors were observed to promote ACO activity and ethylene biosynthesis. *S*-nitrosylated proteomics and *in vitro* biotin-switch assays uncovered that Cys172 in SlACOh4 protein underwent *S*-nitrosylation and increased activity by NO. In addition, NO also activated the expression of *SlACO4h*. Further silencing *SlACOh* confirmed that *S*-nitrosylation of SlACO4 by NO was a key regulation for salt tolerance in tomato plant [52]. These findings expand a new layer of modification on ethylene biosynthesis enzymes for manifesting ethylene biosynthesis.

Methylglyoxal (MG) is a by-product of many physiological processes including respiration, well known for its detrimental effects on cell growth. MG is considered to be a glycating agent which modifies biological macromolecules including proteins to render dysfunction. MG content was high in unripe tomato fruit, but its content decreased despite a climacteric respiration during tomato fruit ripening, suggesting an intimate metabolizing pathway of MG in fruit [91]. A recent study uncovered that MG metabolism was critical for the completeness of tomato fruit ripening, and an overaccumulation of MG in fruit inhibited fruit ripening via suppressing ethylene biosynthesis [91]. It was observed that exogenous MG treatment suppressed fruit ripening and ethylene production. Downregulating *SlGLYI4*, a gene that encoded a key enzyme involved in MG metabolism, disrupted fruit ripening process. Interestingly, the phenotype of *SlGLYI4* silencing fruit could be restored by ethylene or ACC treatment, but not methionine [91]. It was proposed that MG likely could glycate and suppress methionine or SAM biosynthesis enzymes; however, the molecular mechanism remains to be further validated.

## Influences of external or internal stimuli on negative regulators

The expression or protein levels of negative regulators involved in ethylene biosynthesis dynamically alter in response to both external and internal stimuli, finely tuning ethylene production in various physiological processes (Fig. 1, Table 7). These stimuli include ripening and growth signals, hormones, and external environmental cues such as light, abiotic stresses and chemicals. As for ripening/growth signals, the expression of a bunch of negative regulators fluctuate, with most showing a decline as processes undergo. For example, in tomato fruit, the amount of *SlAP2a* transcripts peaked a few days after breaker stage and then decreased in later ripening stages [54]. Another study reported that *SlAP2a* expression was elevated around the mature green stage and continued at high levels until the red fruit stage [55]. Similarly, the transcripts of *SlMADS1* and *SlMADS31* were abundant in green fruit but rapidly declined as ripening progressed [48, 57]. In ripening pear fruit, the expression of *PbRAD23C/D.1* was downregulated [84]. In banana fruit, *MaMPK14* transcript was shown to decrease gradually during ripening [77], and the protein abundance of MaMYB4 and MaXB3 decreased from early stage to late stage during banana fruit ripening [40, 81]. In petunia carpels, there was a rapid increase in the expression of *PhGRL2* and then a decrease in the late stage of flower senescence [25]. On the contrary, albeit they functioned as negative regulators for ethylene biosynthesis, the expression some regulators increased in ripening fruit. For instance, the expression of *SlJMJ7* and *SlDCD2* increased substantially during tomato fruit ripening [42, 90]. The expression of negative regulators could be regulated by internal signaling cascades that triggered as fruit ripening processes. In tomato, the expression of *SlAP2a* was promoted by transcription factor SlVAHOX1 [76]. In banana, *MaXB3* was transcriptionally controlled by ripening regulator MaNAC1/2 [69]. These studies illustrate that certain ripening signals are responsible for regulating the expression of negative regulators to mediate ethylene production during the ripening process.

**Table 7:** The influences of external or internal stimuli on the negative regulators of ethylene biosynthesis.

**Stimulus**	**Negative regulator**	**Species**	**Expression or protein abundance changing pattern**	**Physiological process**	**Reference**
Ripening/growth signal	*SlAP2a*	Tomato	Transcripts peaked several days after breaker stage and continuously declined, or elevated from the mature green stage until to the red stage, and promoted by SlVAHOX1	Fruit ripening	[54, 55, 76]
	*SlMADS1/3*	Tomato	Expression was abundant at green stage and declined as fruit ripen	Fruit ripening	[48, 57]
	*SlJMJ7*	Tomato	Expression increased substantially during fruit ripening	Fruit ripening	[42]
	*SlDCD2*	Tomato	Expression increased substantially during fruit ripening	Fruit ripening	[90]
	*PbRAD23C/D.1*	Pear	Expression decreased during ripening	Fruit ripening	[84]
	*MaMPK14*	Banana	Transcript decreased gradually during fruit ripening	Fruit ripening	[77]
	*MaMYB4, MaXB3*	Banana	Protein abundance decreased during ripening, and MaXB3 was transcriptionally regulated by MaNAC1/2	Fruit ripening	[40, 69, 81]
	*PhGRL2*	Petunia	Expression decreased in senescent flower	Petal senescence	[25]
Hormone	*MaRTH1*	Banana	Expression was inhibited by ethylene	Fruit ripening	[70]
	*MdERF2*	Apple	Protein level was suppressed by ethylene	Fruit ripening	[36]
	*PhGRL2*	Petunia	Expression was influenced by ethylene	Petal senescence	[25]
	*DcNAP2*	Carnation	Expression was significantly induced by ethylene	Petal senescence	[26]
	*SlGASA1*	tomato	Expression was upregulated by gibberellin	Fruit ripening	[82]
	*DkBZR1/2*	Persimmon	Expression increased and protein dephosphorylated in response to epibrassinolide	Fruit ripening	[39]
Light	*MdHY5*	Apple	Expression was upregulated by LED white light	Fruit ripening	[37]
	*PuHY5*	Pear	Expression was upregulated by blue light	Fruit ripening	[41]
	*CmPIF8*	Melon	Expression decreased under far-red light	Fruit ripening	[65]
(A)biotic stress	*CmPIF8*	Melon	Expression was downregulated under *Podosphaera xanthii* infection	Stress response	[65]
	*Sl-miR164a/6*	Tomato	Transcript decreased under cold treatment	Stress response	[30]
	H_2_S	Tomato	Content elevated under osmotic stress	Stress response	[51]
Chemical	*MdERF4*	Apple	Expression was promoted in response to H_2_O_2_	Fruit ripening	[38]
	*MdCDPK7*	Apple	Expression was significantly upregulated by CaCl_2_	Fruit ripening	[49]
	*MdHDT3*	Apple	Expression was significantly inhibited by sodium butyrate	Fruit ripening	[73]

*Cucumis melo* (melon); *Dianthus caryophyllus* (carnation); *Diospyros kaki* (persimmon); *Litchi chinensis* (litchi); *Musa acuminata* (banana); *Malus domestica* (apple); *Petunia hybrida* (petunia); *Prunus persica* (peach); *Pyrus bretschneideri* (pear); *Pyrus ussuriensis* (pear); *Solanum lycopersicum* (tomato).

A vast array of negative regulators are hormone responsive (Table 7). As for ethylene, *MaRTH1* expression in banana fruit was strongly inhibited by ethylene treatment [70]. The protein level of MdERF2 was suppressed by ethylene during ripening of apple fruit [36]. In petunia flower senescence, *PhGLR2* expression also showed a feedback response to ethylene treatment [25]. During carnation petal senescence, *DcNAP2* was significantly induced by ethylene, functioning as a ‘brake’ to retard the activation of ethylene biosynthesis genes by its allele, DcNAP1 [26]. Some negative regulators were gibberellin and epibrassinolide responsive. For example, *SlGASA1* in tomato fruit was upregulated by exogenous GA_3_ treatment and inhibited by GA biosynthesis inhibitor paclobutrazol [82]. During persimmon fruit ripening, the expression of *DkBZR1/2* increased in response to epibrassinolide treatment. Additionally, epibrassinolide treatment promoted dephosphorylation of both DkBZR1 and DkBZR2 proteins [39].

Distinct light sources have also been shown to differentially affect ethylene production in horticultural plants. In fruit, LED white light and blue light irradiation inhibited ethylene production [37, 41], while in melon seedlings, far-red light promoted ethylene production [65]. Both HY5 and PIFs are crucial components in light signaling pathways. MdHY5 and PuHY5 functioned as direct repressors of *ACS1* genes for ethylene biosynthesis, and their expression were upregulated in response to LED white light or blue light (Table 7) [37, 41]. CmPIF8 suppressed *CmACS10* expression and inhibited the positive function of CmERF27 on ethylene production. The expression of *CmPIF8* was downregulated under far-red light irradiation [65].

Biotic and abiotic stresses have an influence on the expression of negative regulators for ethylene biosynthesis (Table 7). Ethylene is an important hormone for stress resilience and its production generally increases in response to stresses, such as powdery mildew infection [92, 93]. In melon, the expression of a negative regulator for ethylene biosynthesis, *CmPIF8*, was downregulated when exposed to *Podosphaera xanthii* infection, thereby releasing the inhibitory effect of CmPIF8 on ethylene biosynthesis [65]. In tomato plant, Sl-miR164a/b was a negative regulator for ethylene biosynthesis via directly targeting *SlNAM* transcripts under cold stress. The level of *Sl-miR164a/b* was downregulated when tomato plant were exposed to cold treatment, which facilitating the enhancement of ethylene production and stress adaption [30]. In tomato plant, H_2_S persulfidated and inhibited the activity of LeACO1 and LeACO2, playing a negative role in ethylene biosynthesis. H_2_S content was observed to be elevated in guard cells under osmotic stress. H_2_S was required for ethylene-triggered stomatal closure to cope with water deficiency while H_2_S negatively control ethylene biosynthesis [51]. These studies collectively highlight an intricate regulation on ethylene biosynthesis by these negative regulators for stress resilience.

External chemical molecules can also regulate the expression of negative regulators for ethylene biosynthesis (Table 7). A low dose of H_2_O_2_ was shown to maintain postharvest apple fruit firmness by suppressing ethylene biosynthesis, in which process MdERF4 acted negatively via directly suppressing *MdACS1* expression. The expression of *MdERF4* was promoted in response to H_2_O_2_ treatment [38]. Exogenous CaCl_2_ suppressed ethylene biosynthesis and apple fruit ripening, and the expression of *MdCDPK7*, a core negative regulator, was significantly upregulated by CaCl_2_ supplement [49]. Sodium butyrate, a potent histone deacetylase inhibitor, promoted ethylene production apple fruit ripening [73]. In this process, MdHDT3 functioned as an epigenetic regulator for suppressing *MdACS1* and *MdACO1* expression. The expression of *MdHDT3* was significantly inhibited by sodium butyrate treatment to induce ethylene production and fruit ripening [73].

## Conclusions and perspectives

Ethylene is pivotal for diverse physiological processes, such as fruit ripening and elongation, flower development and senescence, and responses to stresses. Despite the importance, ethylene production is attenuated to be at optimal levels prior to and after its action, with negative regulators playing significant roles. Over the last decade, the identify of numerous negative regulators, including transcription factors, membrane or enzyme proteins, E3 ligases, miRNAs, kinases and epigenetic regulators, that directly or indirectly suppress ethylene biosynthesis, significantly expands our understanding about the intimate regulation on ethylene biosynthesis in horticultural plants. Despite these advances, there are still many negative regulators identified in the model plants like *Arabidopsis* whose conserved function in inhibiting ethylene biosynthesis await to be explored in horticultural plants. For example, FERONIA receptor kinase directly interacted with SAM synthetase and suppressed its activity for ethylene biosynthesis [94]. ABI1, a negative regulator of abscisic acid signaling, interacted with ACS6 and dephosphorylated its C-terminal fragment, restricting the activity of ACS6 [95]. Abscisic acid and ethylene have a close interaction both in *Arabidopsis* and horticultural plants. In *Arabidopsis*, abscisic acid downstream transcription factor ABI4 could transcriptionally repress the expression of *ACS4* and *ACS8* [96]. Abscisic acid could also activate calcium-dependent protein kinases to phosphorylate ACS6 protein and increase its stability [97]. It unravels complex layers of regulation on ethylene biosynthesis by negative and positive regulators. With the increasing knowledge, the interaction between abscisic acid and ethylene, especially in controlling fruit ripening, will be more comprehensively understood. How does a plant coordinate the complex network of numerous negative and positive regulators from distinct regulatory machinery also is a fascinating question. As more negative regulators to be discovered, a more comprehensive picture of how ethylene biosynthesis is regulated would be expected, which lays significant implications for the growth and development of horticultural plants.

## Data Availability

Not applicable to this review.
